# Association of predicted 10 years cardiovascular mortality risk with duration of HIV infection and antiretroviral therapy among HIV-infected individuals in Durban, South Africa

**DOI:** 10.1186/s13098-019-0502-2

**Published:** 2019-12-16

**Authors:** Olamide O. Todowede, Benn Sartorius, Nombulelo Magula, Aletta E. Schutte

**Affiliations:** 1 Public Health Medicine, School of Nursing and Public Health, University of KwaZulu-Natal, Durban, 4001 South Africa; 2 Department of Health Metrics Sciences, School of Medicine, University of Washington, Seattle, USA; 3 Department of Internal Medicine, Nelson R. Mandela School of Medicine, University of KwaZulu-Natal, Durban, South Africa; 4 Faculty of Infectious and Tropical Medicine, London School of Hygiene and Tropical Medicine, London, UK; 5 Hypertension in Africa Research Team, North-West University, Potchefstroom, South Africa; 6 Medical Research Council Unit for Hypertension and Cardiovascular Disease, North-West University, Potchefstroom, South Africa

**Keywords:** HIV, Cardiovascular risk score, Metabolic syndrome, Antiretroviral therapy

## Abstract

**Background:**

South Africa has the largest population of human immunodeficiency virus (HIV) infected patients on antiretroviral therapy (ART) realising the benefits of increased life expectancy. However, this population may be susceptible to cardiovascular disease (CVD) development, due to the chronic consequences of a lifestyle-related combination of risk factors, HIV infection and ART. We predicted a 10-year cardiovascular mortality risk in an HIV-infected population on long-term ART, based on their observed metabolic risk factor profile.

**Methods:**

We extracted data from hospital medical charts for 384 randomly selected HIV-infected patients aged ≥ 30 years. We defined metabolic syndrome (MetS) subcomponents using the International Diabetes Federation definition. A validated non-laboratory-based model for predicting a 10-year CVD mortality risk was applied and categorised into five levels, with the thresholds ranging from very low-risk (< 5%) to very high-risk scores (> 30%).

**Results:**

Among the 384 patients, with a mean (± standard deviation) age of 42.90 ± 8.20 years, the proportion of patients that were overweight/obese was 53.3%, where 50.9% had low high-density lipoprotein (HDL) cholesterol and 21 (17.5%) had metabolic syndrome. A total of 144 patients with complete data allowed a definitive prediction of a 10-year CVD mortality risk. 52% (95% CI 44–60) of the patients were stratified to very low risk (< 5%) compared to 8% (95% CI 4–13) that were at a very high risk (> 30%) of 10-year CVD mortality. The CVD risk grows with increasing age (years), 57.82 ± 6.27 among very high risk and 37.52 ± 4.50; p < 0.001 in very low risk patients. Adjusting for age and analysing CVD risk mortality as a continuous risk score, increasing duration of HIV infection (p = 0.002) and ART (p = 0.007) were significantly associated with increased predicted 10 year CVD mortality risk. However, there was no association between these factors and categorised CVD mortality risk as per recommended scoring thresholds.

**Conclusions:**

Approximately 1 in 10 HIV-infected patients is at very high risk of predicted 10-year CVD mortality in our study population. Like uninfected individuals, our study found increased age as a major predictor of 10-year mortality risk and high prevalence of metabolic syndrome. Additional CVD mortality risk due to the duration of HIV infection and ART was seen in our population, further studies in larger and more representative study samples are encouraged. It recommends an urgent need for early planning, prevention and management of metabolic risk factors in HIV populations, at the point of ART initiation.

## Background

The introduction of antiretroviral therapy (ART) in the management of human immunodeficiency virus (HIV) infection has resulted in increased life expectancy of infected patients, altering HIV pathogenesis from an acute to a chronic disease [1]. Despite this recognizable success of ART, there are still challenges faced by people living with HIV, such as increased prevalence of metabolic and cardiovascular abnormalities such as lipid abnormalities, diabetes and hypertension [2, 3]. These conditions can occur individually, but more often occur as a cluster of cardiovascular disease (CVD) risk factors, known as metabolic syndrome (MetS), which increases the risk of CVD above what is anticipated for any individual risk factor [4–8].

HIV-infected individuals have a twofold higher risk of CVD morbidity and mortality compared to their uninfected counterparts [9–11]. The main factors leading to these events include HIV infection, inflammation and autoimmune response, ART and the high cardiovascular risk profile of HIV-infected patients [12–14]. The mechanisms of these factors to induce cardiovascular risk are not clear, although an interplay between factors and their complexities have been suggested [15]. CVD risk varies by demographic and clinical factors of the HIV infected populations, for instance some studies showed that severe co-infection increased the risk of CVD in their population while ART adherence and high CD4 count reduce the risk [16, 17]. Other studies indicated the role of protease inhibitors and abacavir, HIV associated inflammation and immune activation to accentuate the risk of CVD among HIV infected populations [18, 19]. Moreover, lifestyle-related CVD risk factors such as diet, obesity, alcohol intake and smoking, common among the general population, further compound the susceptibility of people living with HIV to increased metabolic disturbance [14, 20]. Corroborating evidence indicates increased CVD mortality among people with individual and clustered risk factors (MetS), especially in men and older populations [21, 22].

A systematic review and meta-analysis conducted among HIV-infected populations in sub-Saharan African (SSA) countries indicated a strong association between the use of ART and CVD mortality risk [23]. Similarly, another meta-analysis suggested HIV infection is a consistent risk factor for CVD outcomes, though this varied by geographical location [12]. The association of ART and HIV infection with increased CVD risk has been mostly documented in high-income countries (HIC). However, there is paucity of data reporting this in HIV-hyperendemic setting and in many low-and-middle-income countries (LMICs) [24]. A study in Uganda estimated the 10-year absolute risk of cardiovascular diseases (CVD) among HIV infected populations who were on ART [25]. The association between HIV and CVD risk could be modified in LMIC settings due to other contributors occurring in many of them, such as rapid nutritional, early onset of HIV infection and ART uptake and epidemiological transitions.

Therefore, understanding the potential CVD burden in a hyperendemic HIV context is important to inform routine clinical care, as well as for its prevention and management by clinicians [26, 27]. We therefore assessed the burden of metabolic risk factors and predicted 10-year CVD mortality risk in an HIV-infected population on long-term ART. We further evaluated the relationship between duration of HIV infection and ART use and the predicted 10-year CVD mortality.

## Methods

### Aim of the study

The aim was to assess the burden of metabolic risk factors and predict 10-year CVD mortality risk in an HIV-infected population on long-term ART. We showed the relationship between the duration of HIV infection and ART on cardiovascular mortality risk.

### Study sample

This study is a cross-sectional study (medical chart review) of HIV-infected patients attending Addington Hospital’s antiretroviral clinic an urban setting in Durban, South Africa, from 2004 to 2017. We extracted data from 384 patient files, selected using a simple random sampling approach from a total population of ~ 3800 unique patient files available over this period. A patient was deemed eligible if they attended at least one follow-up visit after ART initiation and was aged ≥ 30 years. These criteria are supported by the South African guidelines for the management of HIV/AIDS, which indicate that HIV-infected patients should have at least two follow-up visits per year [28]. A non-laboratory-based approach was employed for estimating 10-year CVD mortality risk among our HIV-infected cohort, this was implored as a result of missing total cholesterol measurement from most patients charts at the facility. Evidence suggests that the use of body mass index (BMI) instead of total cholesterol levels gives a similar or better prediction of CVD risk estimation, especially in low resource settings with limited access to laboratory testing [29, 30]. The model incorporates the following risk factors in the prediction: age, BMI, current smoker, history and or presence of diabetes, history of blood pressure treatment and systolic blood pressure [30]. The predicted CVD mortality risk was analyzed as a continuous and categorical variable, and the categorized variant was based on the following conventional thresholds: ‘very low’ (< 5%), ‘low’ (5–9.9%), ‘moderate’ (10–19.9%), ‘high’ (20–29.9%), and ‘very high’ (> 30%) [31, 32]. Ethical approval for this study was obtained from the Biomedical Research Ethics Committee (BREC), reference number (BE437/16), at the University of KwaZulu-Natal.

### Data collection

Data were extracted from patient charts using a standardized instrument that was pilot tested. The following information was extracted: demographic—age, sex, race, place of residence; risk factors—smoking, adherence to ART treatment, past and present tuberculosis diagnosis, blood pressure readings; and anthropometric—baseline BMI, height and weight. We also extracted blood profile results for fasting plasma glucose and lipid laboratory data (serum HDL cholesterol level, total cholesterol, serum LDL cholesterol, triglycerides); dates of HIV-infection confirmation and ART initiation; and records of present treatment of hypertension and type 2 diabetes. Other comorbidities (known and recorded presence of non-communicable diseases in the patient’s chart) were extracted when available. If available, data were tracked for all patients at baseline and for at least one or more subsequent follow-up visit at the time of this chart audit. Information on the current and past ART regimen per patient was also extracted. Data captured on the same date as ART initiation are classified as the patient’s baseline record in this study and ordered sequentially for subsequent visits based on the date of presentation. Data were double-entered into an Epi Info 7 database with built-in validation checks.

### Metabolic risk factor classification

We used the cutoff for raised fasting plasma glucose of ≥ 100 mg/dL or 5.6 mmol/L or reported diagnosis and treatment for type 2 diabetes. Similarly, high blood pressure was referred to as systolic pressure of ≥ 130 mmHg and/or a diastolic pressure of ≥ 85 mmHg or reported diagnosis and treatment for hypertension. Other risk factors were defined as raised triglycerides > 150 mg/dL (1.7 mmol/L), reduced HDL cholesterol (< 40 mg/dL (1.03 mmol/L) in men and < 50 mg/dL (1.29 mmol/L) in women) or specific treatment for this lipid abnormality. These definitions are incorporated using the International Diabetes Federation (IDF) definitions for MetS [33]. Body mass index (BMI) was classified into the following three categories: normal < 25 kg/m^2^, overweight 25–29.9 kg/m^2^, and obese ≥ 30 kg/m^2^.

### Data analysis

Data were analysed using STATA version 13.0 [34]. The mean and standard deviation (SD) or median (25th–75th percentiles) were calculated for continuous variables. Difference between means was assessed using the Student’s *t*-test or Wilcoxon rank-sum test (also known as the Mann-Whitney *U* t*est*), if the normality assumption was not upheld. The Pearson Chi square (χ^2^) test was used to measure the strength of linear relationship or the Fisher’s exact test (if fewer than 5 expected observations in all cells in the contingency table) was used to test the association between categorical variables. The correlation between the predicted CVD risks was assessed using the Spearman correlation; whereas the differences in the correlation coefficients across the various settings were tested using the Steiger’s Z test. All statistical tests were assessed at a 5% significance level.

## Results

### Characteristics of the study participants

**Fig. 1:**
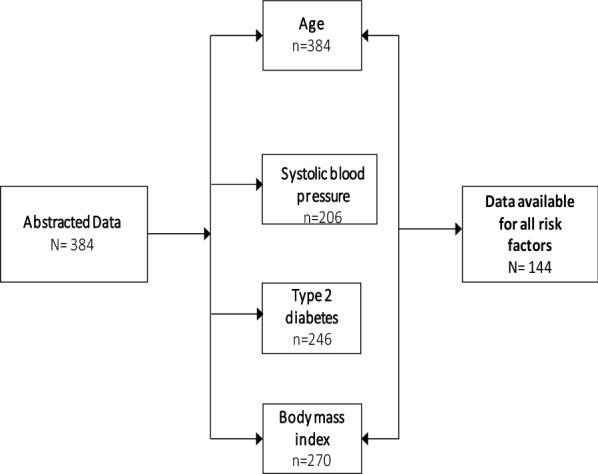
Flow chart of study participants with updated records

A total of 384 randomly selected patient charts were included in this study, with the socio-demographic description of individual risk factors presented in Table 1. However, only 144 patients had complete chart records on all the risk factors required for the assessment of their CVD mortality risk (Fig. 1). Of the 384 patient charts, we observed a mean age of 42.9 (SD8.2) years, 256 (66.3%) were female, and 353 (91.7%) were Black African. The age distribution of the patients are 30–34 (16.15%), 35–39 (27.08%), 40–44 (21.88%), 45–49 (14.58%), 50–59 (4.17%), 60–64 (3.13%) and 1.30% were aged 65 years and above. The mean duration of participants confirmed to be HIV infected was 6.3 ± 4 years. Overall, 201 (52.1%) and 107 (27.7%) participants had, or were presently taking, the first- and second-line ART, respectively, with only two patients on third-line therapy. However, 76 (19.7%) had a missing record of the past or present class of regimen. Tuberculosis infection was the highest comorbidity, with 157 (45%) patients having been previously infected. The most common noncommunicable disease (NCD) comorbidities among participants were 30 (7.5%) with hypertension, 22 (5.7%) with renal impairment, 14 (3.6%) with asthma and there were no cases of lipodystrophy. Overall, 270 patients had a useable baseline BMI measurement, with a mean BMI of 26.5 ± 6.8 kg/m^2^. Women [54 (29.7%)] were significantly more overweight than men [9 (8%); p < 0.001] and 61 (33.5%) women versus 21 (23.6%) men [p < 0.001] were obese.

**Table 1:** Characteristics of the study participants (N = 384)

Characteristics	N	All patients (mean ± SD)	Men (mean ± SD)	Women (mean ± SD)	P-value ^i^
Age (years)	384	42.82 (8.3)	43.26 (8.2)	42.54 (8.4)	0.121
HIV infection duration (years)	220	6.33 (3.9)	5.78 (4.0)	6.72 (3.9)	0.047*
ART duration (years)	383	6.18 (3.43)	5.82 (3.5)	6.39 (3.4)	0.094^*#*^
CD4 cell counts (cells/µL)	364	407.5 (254.6)	347.9 (207.2)	437.9 (271.0)	0.005**
Height (metres)	270	162.7 (9.2)	169.1 (9.6)	159.5 (7.2)	< 0.001**
Weight (Kg)	377	70.8 (17.0)	69.1 (14.0)	71.7 (18.3)	0.324
BMI (kg/m^2^)	270	26.5 (6.8)	23.7 (5.7)	27.9 (6.8)	< 0.001**
Systolic blood pressure (mmHg)	206	120.3 (19.8)	129.7 (25.7)	118.2 (17.3)	0.052^#^
Diastolic blood pressure (mmHg)	207	80.6 (55.0)	79.8 (18.1)	80.9 (63.5)	0.191
Blood glucose (mmol/L)	246	5.6 (2.4)	5.8 (2.4)	5.5 (2.5)	0.012*
Triglycerides (mmol/L)	111	1.6 (1.1)	1.9 (1.4)	1.5 (0.9)	*0.096*^*#*^
HDL cholesterol (mmol/L)	112	1.3 (0.4)	1.2 (0.3)	1.3 (0.4)	0.194

^i^: Wilcoxon rank-sum test; **: significant at 1% level; *: significant at 5% level; ^#^: significant at 10% level

### Prevalence of metabolic syndrome and metabolic risk factors

The prevalence of individual metabolic risk factors of the study participants at baseline (i.e. treatment initiation) is shown in Table 2. Of the 270 patients with a valid BMI classification, 144 (53.2%) were overweight or obese. Among the 206 patients with a blood pressure measurement taken at baseline, most were classified in the normal blood pressure range, at 163 (79.1%). Similarly, among the 246 patients with blood glucose records and the 111 patients with triglyceride records, 74% were classified in the normal blood glucose range and 66.7% had normal triglyceride levels respectively. An almost equal proportion of the patients with HDL cholesterol records presented with both low HDL cholesterol and a normal range of the good cholesterol [57 (50.9%) vs 55 (49.1%); p < 0.010]. Among the 384 participants, there were 165 that could be reliably classified using the IDF MetS definitions alluded to previously, due to missing subcomponent data. Among these 165 participants, 21 (17.5%) were classified as having MetS, with higher prevalence in females [19 (90.5%)] than males [2 (9.5%)]. There was no statistically significant difference in the age distribution when stratified by metabolic syndrome classification (p = 0.102), (Additional file 1: Table S3).

**Table 2:** Prevalence of individual risk factors for cardiovascular disease

Characteristic	Range	Overall (384) n (%)	Male (128) n (%)	Female (256) n (%)	p-value
BMI (Kg/m^2^)	Underweight	12 (4.4)	7 (7.9)	5 (2.7)	< 0.001
	Normal	115 (42.4)	53 (59.6)	62 (34.1)	
	Overweight	75 (27.7)	21 (23.6)	54 (29.7)	
	Obese	69 (25.5)	8 (9)	61 (33.5)	
Blood pressure (mmHg)	< 130/80	163 (79.1)	42 (75)	121 (80.7)	0.373
	≥ 130/80	43 (20.9)	14 (25)	29 (19.3)	
Plasma glucose (mmol/L)	< 5.6	182 (74)	54 (69.2)	128 (76.2)	0.247
	≥ 5.6	64 (26)	24 (30.8)	40 (23.8)	
Triglycerides (mmol/L)	< 1.7	74 (66.7)	19 (63.3)	55 (67.9)	0.650
	≥ 1.7	37 (33.3)	11 (36.7)	26 (32.1)	
Low HDL (mmol/L)	Male < 1.03, Female < 1.29	55 (49.1)	9 (29)	46 (56.8)	< 0.01
	Male ≥ 1.03, Female > 1.29	57 (50.9)	22 (71)	35 (43.2)	

### Estimated 10-year cardiovascular mortality risk

**Fig. 2:**
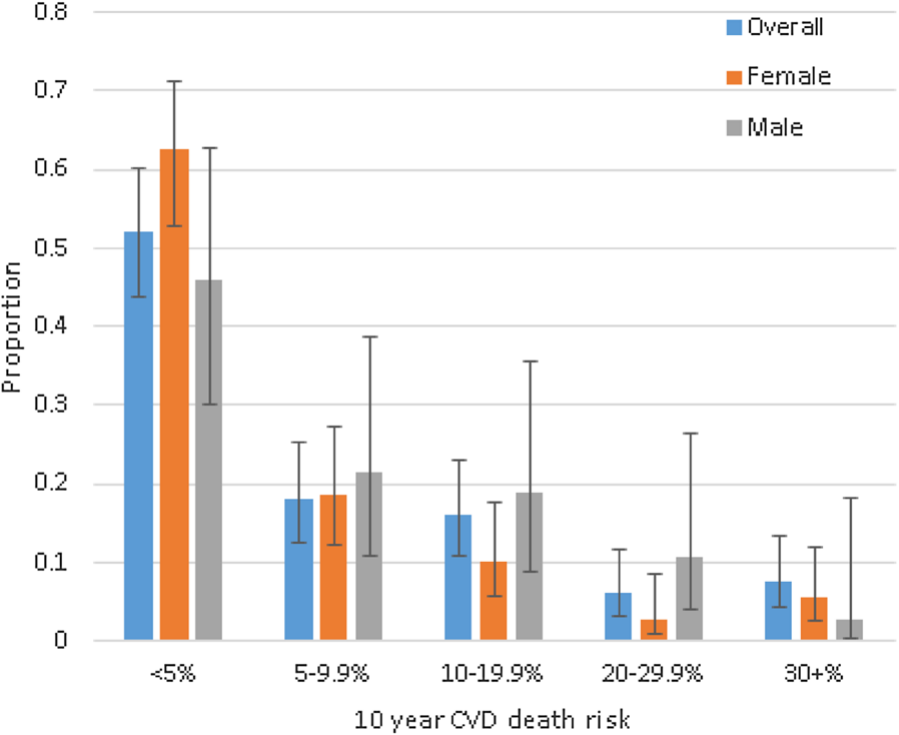
Predicted 10-year CVD death risk stratified by sex

A total of 144 patients had a complete dataset that was modeled for predicting 10-year cardiovascular mortality risk; of these, 107 were females. In this study, the largest proportion of the population belonged in the very low risk group (< 5%) at 52.1% [(75/144, 0.52 (95% CI 0.44–0.60)]. This was 57.0% among females and 37.8% among males (p = 0.136). A significant decrease in the proportion of patients is observed as the prediction moves from very low risk (< 5%) to very high risk (> 30%)—Fig. 2. Approximately 6% [(9/144, 0.06 (95% CI 0.03–0.12)] of this study population were classified in the low risk (20–29.9%) 10-year CVD mortality risk category. Similarly, about 8% [(0.08 (95% CI 0.04–0.13)] of the patients were at a greater than 30% 10-year risk of CVD mortality. However, the stratification of the proportion of our study sample, by the CVD mortality risk scores, was statistically insignificant (Fig. 2 p = 0.136). There was a significant positive association between presence of Mets and 10-year cardiovascular mortality risk category, with 5 (33.33%), p = 0.002 of patients at very high risk had MetS, (Additional file 1: Table S4).

### Duration of HIV infection, antiretroviral therapy and cardiovascular mortality risk

Table 3 displays the estimation of the five categories of non-laboratory-based CVD mortality risk scores compared by age, duration of years of HIV infection, and ART use. We observed an increase in the mean age of our sample from very low-risk (37.52 ± 4.50 years), moderate-risk (48.05 ± 5.64 years), to the very high-risk score band (57.82 ± 6.27 years). The median duration of HIV-infection confirmation among patients who were at a very low CVD mortality risk was 6.64 (3.63–9.86) years, while those at moderate-risk was 6.91 (5.11–8.02) years and the very high-risk was 7.20 (4.49–11.05) years. Nonetheless, a higher proportion of patients who had been on ART for longer than 7.96 ± 4.38 years were in the high-risk score band, compared to the very low-risk 6.52 ± 3.47 years’ band for CVD mortality. This study showed that duration of HIV infection and ART didn’t correlate with the predicted CVD mortality risk categorized thresholds.

**Table 3:** Relationship between age, duration of HIV infection and antiretroviral therapy (in years) and cardiovascular mortality risk score classification

Variables (years)	Risk score					
	< 5.00%	5.00–9.9%	10.00–19.9%	20.00–29.9%	> 30.00%	P-value^i^
Age						
N	75	26	23	9	11	
Mean (SD)	37.52 (4.50)	44.47 (5.52)	48.05 (5.64)	51.67 (4.36)	57.82 (6.27)	Overall: < 0.001
Duration of HIV infection						
N	41	16	12	7	5	
Median (P25–P75)	6.64 (3.63–9.86)	8.11 (4.46–11.07)	6.91 (5.11–8.02)	7.41 (2.20–12.10)	11.51 (7.11–11.69)	0.684
Duration since ART initiation						
N	75	26	23	9	11	
Median (P25–P75)	6.37 (4.44–9.06)	8.26 (4.64–11.01)	6.19 (4.78–7.31)	7.39 (7.11–11.85)	8.09 (6.83–10.55)	0.119

^i^One-way analysis of variance ANOVA with Bonferroni correction for multiple testing

However, analysing the predicted 10-year CVD mortality risk as a continuous risk score and adjusting for patients’ age, increasing duration of ART (p = 0.007) and HIV infection (p = 0.002) were significantly associated with increased patient’s susceptibility to the risk of CVD mortality. (Additional file 1: Table S1).

### The proportion of other related factors with CVD mortality risk

A bivariate analysis (Table 4) of our study sample could not suggest if race was associated with high or very high risk of CVD mortality among our population, as the proportion of Black Africans to other races in our study is not comparable. Patients who had changed from the first-line and were presently on second-line ART regimen [8/70 (11.43%)], were more likely to be classified as very high risk than those on first-line treatment [3/73 (4.11%)], but this was not statistically significant (p = 0.211). Among those with previous tuberculosis infection, there appeared to be a lower likelihood of being at a very high risk of CVD compared to those without previous tuberculosis infection [(3/70 (4.29%) versus 8/72 (11.11%), p = 0.063].

**Table 4:** Bivariate analysis of factors associated with CVD mortality risk

Variables	Total (N = 144)	< 5.00%	5.00–9.90%	10.00–19.90%	20.00–29.90%	> 30.00%	P value
Black Africans	133	71 (53.38)	24 (18.05)	23 (17.29)	9 (6.77)	6 (4.51)	< 0.001^Ϯ^
Other races^α^	11	4.0 (36.36)	2 (18.18)	–	–	5 (45.45)	
1st line regimen	73	37 (50.68)	13 (17.81)	16 (21.92)	4 (5.48)	3 (4.11)	0.211^*^
2nd line regimen	70	37 (52.86)	13 (18.57)	7 (10)	5 (7.14)	8 (11.43)	
Previous tuberculosis	70	44 (62.86)	8 (11.43)	11 (15.71)	4 (5.71)	3 (4.29)	0.063^Ϯ^
No tuberculosis	72	30 (41.67)	18 (25.00)	11 (15.28)	5 (6.94)	8 (11.11)	

^Ϯ^Fisher’s exact test; * Pearson Chi square (χ^2^) test, ^α^ Coloured and White

Analysing 10-year cardiovascular mortality risk as a linear variable using a continuous regression formulation (Bivariate and multivariable adjusted), age (p < 0.001) remained independently associated with 10 year predicted CVD mortality risk. The analyses also suggest an association between African ethnicity (p < 0.001), having previous TB infection (p = 0.014) and being female (p < 0.001) and increased CVD mortality risk.

There was no difference in baseline CD4 when comparing individuals with MetS to those without (median baseline CD4 of 452.5 [IQR: 332–667] versus 390 [IQR: 210–589], p-value = 0.285). Furthermore, median baseline CD4 ranged from 363 (IQR: 227–525) among those classified with < 5% 10-year cardiovascular mortality risk to 389.5 (IQR: 194–508) among those with 30+% 10-year cardiovascular mortality risk (p-value = 0.484).

## Discussion

HIV-infected and treated patients are susceptible to increased CVD risk factors, as well as cardiovascular morbidity and mortality outcomes. Although this susceptibility is associated with traditional risk factors, HIV infection, antiretroviral regimen and dynamics that emanate from the infection may also contribute to increased cardiovascular risk [35–37]. This present study reports the proportion of metabolic syndrome, metabolic risk factors and the associations thereof with the duration of HIV infection and ART. We also predicted the 10-year CVD mortality risk in HIV-infected patients treated for a mean duration of 6.18 years.

The study participants are aged 30 years and above, with the mean age of 42.90 ± 8.20. Most of the patients (45%) have had tuberculosis in the past, which emphasizes the epidemic of HIV/TB co-infection in South Africa [38]. We realised a significantly high prevalence of overweight and obesity among our patients. These were more prevalent among the female participants, and confirms the epidemic of obesity in our setting [39]. Most of our study samples had normal blood pressure (79.1%), though this was a baseline record. There is a probability of increase blood pressure over time among the population that was not monitored, understanding that South Africa has high burden of high blood pressure [40]. Recent study in South Africa among HIV infected cohort showed a high prevalence of hypertension at baseline and about 15% increased prevalence at follow -up after ART initiation [41]. In Denmark, a low prevalence of hypertension was reported in their population [42]. However, this outcomes depends on ART adherence, type of ART use and the study population [41]. Our study found an increased prevalence of MetS (17.5%), especially in African women and supports the known increased high age-standardized CVD mortality rate in South Africa, driven by high blood pressure and obesity [43]. Age-standardized CVD mortality is increasing in sub-Saharan Africa countries, though varies by countries and mostly driven by growing age and population’s health transition from communicable to non-communicable diseases [44–46]. Obesity and overweight, associated with the increasing presence of lipid disorder such as low HDL cholesterol and raised triglycerides, is prevalent in HIV-infected patients [47, 48]. This factors are associated with the development of metabolic disorder and CVD outcomes and this was indicated in our results. Additionally, related studies conducted in some Northern American and Europe reported that people living with HIV with increased age (45–60 years) were more inclined to die from CVD compared to the general population [26, 49], as our study indicates.

We showed that most of the study samples (52%) were at a very low risk of CVD mortality, especially women. In Uganda, a large HIV positive cohort population study, showed that men were at high 10-year risk of acute CVD and ART treated populations were more at risk of longer term CVD, as suggested in our study [25]. Of our study patients, 8% were shown to be at greater than a 30% 10-year risk of CVD mortality, similar to what was reported among a general population (5.7% and above) with a comparable age range in Germany and India [50, 51]. We have shown that there is a linear association between older age, patients who have been on ART for longer, and are in the high-risk score band (7.96 ± 4.38 years) compared to very low risk (6.51 ± 3.47 years) for CVD mortality. Predicted CVD mortality risk was not associated with the duration of treatment or HIV infection using the categorized threshold scores but was independently associated with increased age. Moreover, in a multivariate analysis and adjusting for age, duration of HIV infection and ART were significantly associated with 10 years predicted CVD mortality risk. This result emphasizes the additional effect of HIV related and individual components of MetS on the susceptibility to CVD mortality risk, at the absence of increased age. HIV infection, immune activation and inflammation are related to cardiovascular risk among the population, characterized by the complex interaction of systematic factors at all stages of HIV-infection progression [13, 16]. Likewise, both untreated HIV-infected and treated persons are adversely susceptible to CVD outcomes [19]. Nonetheless, studies have suggested CVD development to be more greatly associated with the duration and type of ART regimen (specific classes) than HIV-infection itself [52–55]. A recent study strengthens this evidence, reporting a high prevalence of metabolic risk factors among infected patients who have been on a protease inhibitor regimen, with 35% cardiovascular mortality at a 30-day endpoint [56, 57]. HIV-infected patients have shown a substantial development of CVD and its outcome progression within three years of ART initiation [58]. Islam et al. [59] showed a yearly increasing CVD risk among treated HIV-infected patients, establishing that the duration of ART exposure is strongly associated with CVD outcome. Višković et al. [13] analyzed the association between the presence of CVD biomarkers and related HIV-infected patient characteristics. The results of their study showed a positive correlation between the duration of HIV infection and ART and the presence of CVD markers, with a median of 8 and 6 years respectively [13]. Though in a Danish population, ageing, duration of ART initiation and HIV infection were not related with the risk of CVD [60]. This present study indicates a linear probability of a 10-year CVD mortality risk among its patients and the relationship between duration of infection or treatment [61].

Several studies have suggested CVD as an increasing cause of mortality in HIV-infected patients [26, 49]. A report shows the existence of a mutual interaction between traditional risk factors and HIV related factors namely HIV infection and long-term use of ART [15]. The traditional factors include high BMI, low HDL, which has shown to be prevalent among this study participants. American Heart Association recently reported that high BMI has shown to be independently associated with significant higher risk of death attributable to CVD [62]. In South Africa, participants with mean age 46 years and mostly African men, were within moderate to high CVD risk, in the presence of increased metabolic factors such as high blood pressure, high blood glucose [63]. Our study relates with literature that CVD death among HIV infected population has increased over the same period AIDS mortality decreased, even after matching and controlling for CVD known risk factors [64]. A huge reduction of CVD mortality is feasible by improving the distribution of risk factors in a population, which is applicable in our participants [65]. Treated HIV patients are clearly at a comparable CVD risk as general population, with the strong influence of aging HIV population. HIV infected population study in Brazil showed that HIV specific risk factors were strongly associated with CVD mortality risk than the traditional risk factors as age [66]. This variation emphasizes the complex interplay of different sets of risk factors for CVD mortality by populations [67]. This underlines the emerging need for an enhanced CVD risk-prediction model for HIV-infected populations, as a CVD mortality prediction tool has been reported to underestimate cardiovascular risk in HIV patients [26, 68, 69]. Studies have argued that the relationship between risks of CVD in HIV populations remains obscure and inconclusive, as the clinical significance of an absolute risk of CVD risk remains low in the HIV-infected population, specifically with certain types of ART [70, 71].

Our study indicated most of the population were at low risk of CVD mortality. Noteworthy is the modest population size used for the estimation, which might not give the true reflection of the general population. Our study suggests that screening of CVD risk factors (as noted by the large number of missing information during the patient record review) occurs less frequently in healthcare practice than is recommended [72]. This resulted in few patients with complete variables for more precise estimation of CVD mortality risk compared to the target study sample size initially Our results are of interest and importance to public health, while taking our limitations into account. We relied on hospital -based patient medical chart reported diagnoses, though these might not be complete and entirely give accurate representation of our population, resulting in underestimation. Most of our subjects were females and of the African race, our results may not be generalizable to other populations. We have used cross-sectional data (chart audit) to predict the relationship between the duration of HIV infection and ART on cardiovascular mortality risk, hence causation cannot be ascertained. We were unable to include an HIV-uninfected control population from the same region to compare the 10-year CVD risk. Moreover, studies among the South African general population have reported a high prevalence of CVD risk factors, ranging between 21.8 and 42.6% specifically among females [73–75]. This study also acknowledges another limitation in that we used the South African national smoking proportion to estimate the prevalence of smoking among our present population. However, this variable does not contribute much weight to our analysis [76]. Our study was not able to incorporate cholesterol concentrations as one of the variables of the CVD mortality risk estimation; however, the use of BMI is equally reliable for the prediction [32]. Nevertheless, we found a high proportion of metabolic risk factors with the possibility of cardiovascular mortality risk in a population of treated and stable HIV-infected patients. Our study recommends the scaling up of the routine monitoring of CVD risk factors during the management of HIV infection and treatment. This is supported by studies that have shown the increased susceptibility of the general population to CVD, including people living with HIV [16, 77].

## Conclusions

In summary, in a South African population infected with HIV for a mean duration of 6 years, we found relationship between duration of infection and duration of ART use with a 10-year predicted CVD mortality risk. However, predicted CVD mortality risk was strongly associated with age, as would also be expected in an uninfected population. Where 1 in 2 participants had increased adiposity, only 7% had hypertension. There was an implication of increased age, HIV infection and ART to the population’s predisposition to a predicted 10-year cardiovascular mortality risk. This implies that treated HIV patients should be as equally considered for CVD risk management as would be uninfected patients. However, modifiable risk factors may also have increased their susceptibility, so further studies are needed to clarify the risk differences between the contribution of traditional risk factors and HIV-related factors. This will assist with planning early prevention and management of metabolic risk factors and cardiovascular disorder outcomes in HIV-infected populations.

## Supplementary information

**Additional file 1: Table S1:** Bivariate linear analysis of CVD Mortality risk. **Table S2.** Multivariable linear analysis of CVD Mortality risk. **Table S3.** Prevalence of Metabolic syndrome by Age distribution. **Table S4.** Prevalence of Metabolic syndrome by CVD mortality risk categories.

## Data Availability

The dataset used and/or analyzed during the current study are available from the corresponding author on reasonable request.

## Abbreviations

HIV: human immunodeficiency virus
CVD: cardiovascular disease
MetS: metabolic syndrome
HDL: high density lipoprotein
SSAs: sub-Sahara African countries
HICs: high-income countries
LMICs: low-and-middle-income countries
