# Supplementary material for: Association of predicted 10 years cardiovascular mortality risk with duration of HIV infection and antiretroviral therapy among HIV-infected individuals in Durban, South Africa
Source: Diabetol Metab Syndr. 2019 Dec 16;11:105. doi: 10.1186/s13098-019-0502-2 (PMC6916025; doi:10.1186/s13098-019-0502-2)
Supplement: Supplementary file 1 — Additional file 1: Table S1: Bivariate linear analysis of CVD Mortality risk. Table S2. Multivariable linear analysis of CVD Mortality risk. Table S3. Prevalence of Metabolic syndrome by Age distribution. Table S4. Prevalence of Metabolic syndrome by CVD mortality risk categories. [file 13098_2019_502_MOESM1_ESM.docx]

**Table S1: Bivariate linear analysis of CVD Mortality risk**

| **Bivariate** | | | | |
| --- | --- | --- | --- | --- |
| **Covariate** | Coef. | [95% Conf. | Interval] | p-value |
| **Age (in Years)** | 0.10 | 0.08 | 0.12 | **<0.001** |
| **Female** | 1.97 | 1.61 | 2.32 | **<0.001** |
| **African** | -0.38 | -1.17 | 0.41 | 0.348 |
| **Duration HIV infection** | 0.11 | 0.04 | 0.18 | **0.002** |
| **Duration ART** | 0.09 | 0.02 | 0.15 | **0.007** |
| **ChangeinFirstRegimen** | 0.26 | -0.16 | 0.69 | 0.217 |
| **PreviousTB** | -0.53 | -0.95 | -0.11 | **0.014** |

| **Multivariable** | | | | |
| --- | --- | --- | --- | --- |
| **Covariate** | Coef. | [95% Conf. | Interval] | p-value |
| **Age (in Years)** | 0.09 | 0.07 | 0.10 | **<0.001** |
| **Female** | -0.09 | -0.47 | 0.29 | 0.635 |
| **African** | 1.91 | 1.68 | 2.13 | **<0.001** |
| **Duration HIV infection** | Excluded due to high collinearity with duration on ART and missing values | | | |
| **DurationART** | 0.01 | -0.02 | 0.04 | 0.418 |
| **ChangeinFirstRegimen** | Excluded due to high collinearity with duration on ART | | | |
| **PreviousTB** | -0.18 | -0.38 | 0.02 | ***0.078*** |
| **_cons** | 19.95 | 19.23 | 20.68 | --- |

**Table S2: Multivariable linear analysis of CVD Mortality risk**

**Table S3: Prevalence of Metabolic syndrome by Age distribution.**

| **Age)years** | **Presence of MetS(n%)** | **Absence of MetS (n%)** | **Total (N%)** |
| --- | --- | --- | --- |
| **30 – 34** | 2 (9.52) | 26 (18.06) | 28 (16.97) |
| **35 – 39** | 4 (19.05) | 36 (25.00) | 40 (24.24) |
| **40 – 44** | 2(9.52) | 33 (22.92) | 35 (21.21) |
| **45 – 49** | 4 (19.05) | 19 (13.19) | 23 (13.94) |
| **50 – 54** | 4 (19.05) | 21 (14.58) | 25 (15.15) |
| **55 – 59** | 2 (9.52) | 5 (3.47) | 7 (4.24) |
| **60 – 64** | 2 (9.52) | 3 (2.08) | 5 (3.03) |
| **≤65** | 1 ( 4.76) | 1 (0.69) | 2 (1.21) |

**Table S4: Prevalence of Metabolic syndrome by CVD mortality risk categories.**

| **CVD risk** | **<5%** | **5-9.9%** | **10-19.9%** | **20-29.9%** | **30+%** |
| --- | --- | --- | --- | --- | --- |
| **Presence of MetS** | 3 (20 ) | 3 (20) | 2 (13.33) | 2 (13.33) | 5 (33.33) |
| **Absence of MetS** | 62 (55.36) | 22 (19.64) | 18 (16.07) | 5 (4.46) | 5 (4.46) |
